# Infant Non-Secretor Histoblood Group Antigen Phenotype Reduces Susceptibility to Both Symptomatic and Asymptomatic Rotavirus Infection

**DOI:** 10.3390/pathogens13030223

**Published:** 2024-03-04

**Authors:** Benjamin Lee, Md Abdul Kader, Masud Alam, Dorothy M. Dickson, Patrick Harvey, E. Ross Colgate, Mami Taniuchi, William A. Petri, Rashidul Haque, Beth D. Kirkpatrick

**Affiliations:** 1 Department of Pediatrics, Vaccine Testing Center and Translational Global Infectious Diseases Research Center, Larner College of Medicine, University of Vermont, Burlington, VT 05405, USA; 2 International Centre for Diarrhoeal Disease Research, Bangladesh, Dhaka 1212, Bangladesh; abdul.kader@nstu.edu.bd (M.A.K.); mohammad-masud.alam@med.uvm.edu (M.A.); rhaque@icddrb.org (R.H.); 3 Department of Microbiology and Molecular Genetics, Vaccine Testing Center and Translational Global Infectious Diseases Research Center, Larner College of Medicine, University of Vermont, Burlington, VT 05405, USA; dorothy.dickson@med.uvm.edu (D.M.D.); patrick.harvey@med.uvm.edu (P.H.); ecolgate@emmes.com (E.R.C.); beth.kirkpatrick@med.uvm.edu (B.D.K.); 4 Division of Infectious Diseases and International Health, University of Virginia, Charlottesville, VA 22903, USA; mt2f@virginia.edu (M.T.); wap3g@virginia.edu (W.A.P.J.)

**Keywords:** rotavirus, diarrhea, gastroenteritis, secretor, FUT2, histoblood group antigen

## Abstract

The infant non-secretor histoblood group antigen phenotype is associated with reduced risk of symptomatic rotavirus diarrhea, one of the leading global causes of severe pediatric diarrheal disease and mortality. However, little is known regarding the role of secretor status in asymptomatic rotavirus infections. Therefore, we performed a nested case–control study within a birth cohort study previously conducted in Dhaka, Bangladesh, to determine the association between infant secretor phenotype and the odds of asymptomatic rotavirus infection, in addition to the risk of rotavirus diarrhea, in unvaccinated infants. In the parent cohort, infants were enrolled in the first week of life and followed through the first two years of life with multiple clinic visits and active surveillance for diarrheal illness. Secretor phenotyping was performed on saliva. Eleven surveillance stools collected over the first year of life were tested for rotavirus by real-time RT-PCR, followed by conventional PCR and amplicon sequencing to identify the infecting P-type of positive specimens. Similar to findings for symptomatic diarrhea, infant non-secretors experienced significantly fewer primary episodes of asymptomatic rotavirus infection through the first year of life in a likely rotavirus P-genotype-dependent manner. These data suggest that non-secretors experienced reduced risk from rotavirus due to decreased susceptibility to infection rather than reduced infection severity.

## 1. Introduction

Infections due to Group A rotaviruses (RVs) remain one of the leading causes of severe pediatric gastroenteritis in low- and middle-income (LMIC) settings, responsible for up to 200,000 diarrheal deaths yearly among children < 5 years [1,2,3,4]. This occurs despite the availability of live-attenuated oral RV vaccines (LORVs), both due to ongoing access constraints and the challenge of oral vaccine underperformance in these populations, with reduced clinical effectiveness of LORVs observed in LMICs compared to high-income settings [5]. The causes for LORV underperformance are multifactorial, but a common theme is the involvement of factors that impact the gut microenvironment and subsequent mucosal immune responses [1,6,7,8].

Rotaviruses are non-enveloped viruses containing a segmented double-stranded RNA genome within a triple-layered capsid. Traditionally, RVs have been classified using a binary nomenclature according to the serotypes or genotypes of the two outer capsid layer proteins: VP7 forms the outer glycoprotein shell (G-type), and VP4 is a protease-sensitive spike (P-type) that protrudes from the surface of the virion and is believed to mediate attachment to host enterocytes following proteolytic cleavage by intestinal trypsin [9]. For example, the most commonly used LORV worldwide, Rotarix (GlaxoSmithKline) [10], is an attenuated G1P[8] strain. 

Numerous studies from multiple settings have convincingly demonstrated that non-secretors are at lower risk of RV diarrhea, mainly due to reduced susceptibility to symptomatic infection with P[8] and/or P[4] rotaviruses, as reviewed elsewhere [11]. An individual’s secretor status denotes a specific histoblood group antigen (HBGA) phenotype, which is genetically determined by *FUT2*, a gene that encodes an α-[1,2]-fucosyltransferase responsible for the expression of specific 2-fucosylated oligosaccharides on mucosal surfaces and bodily secretions, including the gut mucosa and in breast milk in the form of human milk oligosaccharides (HMOs). Individuals with functional enzyme are termed “secretors”, while those with inactivating *FUT2* mutations (generally in the form of single-nucleotide polymorphisms) are termed “non-secretors” and express a different glycan array in these body compartments [12]. It is believed that various pathogens, including rotaviruses and other enteric viruses, utilize specific oligosaccharide ligands as potential cellular receptors in a type-specific manner, leading to differential susceptibility to infection based on the specificities of the glycans expressed in the gut according to underlying host genetic status [13,14].

Because of the association of secretor status with susceptibility to rotaviruses, likely in a P-genotype-dependent manner, numerous additional studies have examined whether this could influence the vaccine take of various LORVs, as reviewed elsewhere [15]. However, there are few data on whether secretor status also influences the risk of asymptomatic infection with wild-type (i.e., unattenuated) rotaviruses in young children. Therefore, we conducted a nested case–control analysis within the Performance of Rotavirus and Oral Polio Vaccines in Developing Countries (PROVIDE) birth cohort study, conducted in Dhaka, Bangladesh, to investigate whether previously observed associations between infant secretor status and risk of RV diarrhea extended to risk of asymptomatic wild-type infections as well.

## 2. Materials and Methods

### 2.1. Study Population

This was a nested case–control study performed within the PROVIDE birth cohort study, performed in Dhaka, Bangladesh, from 2011 to 2014 [16]. Full study results have been published [17,18]. In the parent study, 700 infants were enrolled in the first week of life and followed through age 2. Infants were randomized 1:1 to receive either Rotarix or no vaccine at 10 and 17 weeks of age in a formal efficacy trial. The PROVIDE study was conducted as a collaboration between the International Centre for Diarrhoeal Disease Research, Bangladesh (icddr, b), the University of Vermont, and the University of Virginia; the study was registered at ClinicalTrials.gov (NCT01375647) and approved by the respective ethical review board of each institution. All participating families signed informed consent prior to study participation.

Each child underwent surveillance stool collection at 11 pre-specified time points through year 1, at 1, 6, 10, 12, 14, 17, 18, 24, 39, 40, and 52 weeks, and all participants underwent active community-based diarrheal surveillance with attempted specimen collection for each diarrheal episode. Episodes of RV diarrhea were diagnosed by stool antigen enzyme immunoassay (EIA; ProSpect, Oxoid, UK).

Following study completion, a subset of infants was selected in a case–control manner for more detailed evaluation of measures of rotavirus infections and immune response. Cases were defined as infants who completed the study per protocol through year 1, had residual specimens available for analysis, and experi-enced their first episode of RV diarrhea during the primary post-vaccination period (18–52 weeks of life). Cases were selected 1:2 from the vaccinated and unvaccinated groups, re-spectively. Two matched controls, defined as infants who remained RV diarrhea-free through the end of year 1, were selected for each case.

### 2.2. Detection of Asymptomatic Rotavirus Infections and P-Type Confirmation

To detect asymptomatic RV infections, stored surveillance stool specimens from all case–control participants underwent total nucleic acid extraction using the QIAamp Fast DNA stool Mini Kit (QIAGEN, Hilden, Germany) as previously described [19]. TNA extracts were tested for RV infection by real-time PCR, followed by conventional PCR amplification of the VP8* gene segment for the P-type identification of positive specimens via Sanger sequencing, as previously described [20]. Diarrheal specimens positive for RV antigen underwent the identical protocol for P-type determination.

### 2.3. Secretor Status Phenotyping

All infants had previously undergone Lewis antigen and secretor status phenotyping on stored saliva specimens using a combination of Lewis a and b antigen dot-blot assay and *Ulex europaeus* agglutination EIA, as previously described [21]. Secretor status could be inferred from Lewis status based on the action of both enzymes on the same precursor oligosaccharides: infants with any Lewis b antigen expression were secretors, while those with only Lewis a expression were non-secretors. Among infants negative for both Lewis a and b, positive *U. europaeus* agglutination confirmed secretor status.

### 2.4. Statistical Analysis

For this analysis, we evaluated unvaccinated infants only to determine the role of secretor status on asymptomatic, wild-type RV infection through year 1 of life. The primary outcome was the detection of asymptomatic RV infection, defined as any surveillance specimen positive for RV RNA at a real-time PCR cycle threshold (Ct) cut-off of <34 [21]. Additional sensitivity analysis was performed using a cut-off of <40. Categorical outcomes were analyzed using Chi Square or Fisher’s Exact Test to estimate proportion difference with corresponding odds ratios (ORs) and 95% confidence intervals (CIs). The Mann–Whitney U Test was used to compare continuous outcomes across groups with non-normal distributions. All analyses were performed on SPSS version 29 (IBM, Armonk, NY, USA). Differences were considered statistically significant at the two-sided *p*-value of <0.05.

## 3. Results

From the parent PROVIDE cohort, a total of 405 evaluable infants were identified for inclusion in the case–control group, of whom, 210 were unvaccinated. Among unvaccinated children, N = 169 also had secretor and Lewis phenotyping results available and were included in this analysis. Baseline sociodemographic variables for the evaluated unvaccinated group are presented in Table 1. N = 114 (67%) were secretors and N = 55 (33%) were non-secretors, similar to the parent cohort [21]. A total of N = 86 (51%) had an episode of RV diarrhea during year 1, and N = 83 remained RV diarrhea-free. As previously demonstrated, infant non-secretors were significantly less likely to have an episode of RV diarrhea through year 1 [21], but no differences were noted in any other baseline variable, including Lewis status.

### 3.1. Effect of Secretor Phenotype on Incidence of Asymptomatic Rotavirus Infection

**Figure 1:**
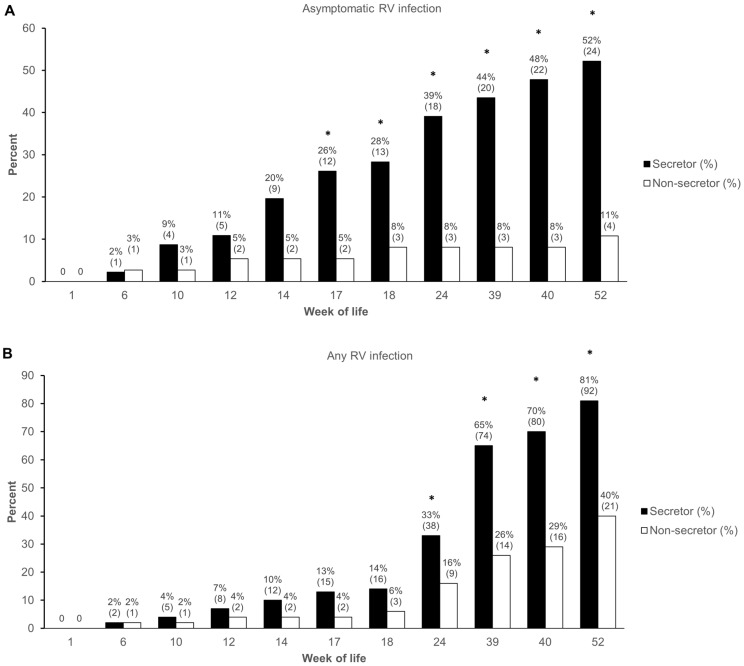
Cumulative incidence of rotavirus infection in unvaccinated children through year 1 of life according to secretor phenotype. Cumulative incidence of (**A**) asymptomatic rotavirus infection in unvaccinated children who remained RV diarrhea-free through year 1 of life, and (**B**) any rotavirus infection (symptomatic RV diarrhea or asymptomatic infection) by each indicated time point. The numbers above each bar indicate the percentage of evaluable children with infection by that time point, along with the absolute number of children with infection by that time point in parentheses. * *p* < 0.05.

Because prior natural infection might mediate subsequent immunity, we first analyzed the effect of secretor phenotype on asymptomatic RV infections in unvaccinated infants who remained RV diarrhea-free through the first year of life (N = 83). Using a cut-off of Ct < 34 to define RV infection, a total of N = 28 (34%) infants had at least one detected episode of asymptomatic RV infection in the first year of life, with non-secretors demonstrating significantly reduced odds for asymptomatic infection by the end of year 1 (OR 0.111, 95% CI 0.034–0.365; Table 2). The cumulative incidence of infection through each time point is depicted in Figure 1A. Because we suspected that asymptomatic infections might demonstrate very low viral loads compared to diarrheal specimens, we conducted an additional sensitivity analysis defining asymptomatic infection as the detection of RV at any Ct < 40. Although the effect was somewhat diminished, the results were generally unchanged and remained significant at the higher cut-off (Table 2). Similarly, the results were unchanged when comparing the effect of secretor phenotype on the odds of any RV infection, either symptomatic RV diarrhea or asymptomatic infection, in all unvaccinated children (N = 169) (Table 2, Figure 1B). Notably, using the most liberal cut-off for defining asymptomatic RV infection (Ct < 40), nearly all secretor infants (92%) demonstrated either symptomatic or asymptomatic RV infection by the end of the first year of life.

Next, we compared viral burden in secretors versus non-secretors who remained RV diarrhea-free through the first year. To achieve this, we identified the lowest Ct value (indicating the highest quantity of virus) in each participant with any detected asymptomatic infection. At a cut-off of Ct < 34, there was no difference in Ct distribution in secretors with any asymptomatic infection (N = 24) compared to non-secretors (N = 4; *p* = 0.84 by Mann–Whitney U test). Using a cut-off of Ct < 40, the Ct distribution was significantly different in secretors (N = 37) compared to non-secretors (N = 21), with secretors having a lower median Ct value than non-secretors (32.7 vs. 36.3, respectively; *p* = 0.012 by Mann–Whitney U test).

Together, these data suggest that non-secretors have a reduced frequency of natural wild-type RV infection (both symptomatic and asymptomatic) and lower viral burden during asymptomatic infections compared to secretors.

### 3.2. P-Type Distribution among Asymptomatic Infections

Next, we assessed whether susceptibility to asymptomatic infection was P-type specific, as has been observed for RV diarrhea. Among children with any asymptomatic infection at any Ct < 40 but who remained RV diarrhea-free through one year, N = 58 had P-type results available for analysis. Similar to results seen previously for RV diarrhea, no P[4] infections were detected in non-secretor children (Table 3; *p* = 0.050). No difference was seen in the risk of asymptomatic P[6] infections. Interestingly, however, asymptomatic P[8] infections were more frequent in non-secretors compared to secretors, although this did not reach statistical significance, likely due to the limited sample size (Table 3; *p* = 0.077). When evaluating all children with either symptomatic RV diarrhea or asymptomatic infection at any Ct < 40 (N = 144), these results were unchanged, with non-secretors having no detected P[4] infections (Table 3; *p* = 9.3 × 10^−5^) but with no differences being seen in the frequency of P[6] or P[8] infections according to secretor phenotype.

## 4. Discussion

In a birth cohort of infants from urban Dhaka, Bangladesh, infant secretor phenotype had a significant impact on the risk of asymptomatic wild-type RV infections. These results confirm that the previously observed protective effect of non-secretor status on symptomatic RV diarrhea [21] extends to asymptomatic wild-type RV infections, with non-secretor infants demonstrating significantly reduced odds for asymptomatic RV infection by the end of the first year of life, including among children who never experienced an episode of symptomatic RV diarrhea. In this high-incidence setting, virtually all secretor infants had experienced at least one RV infection, defined as the real-time PCR detection of RV in stool, by the end of the first year of life.

Overall, these results suggest that non-secretors are protected from RV diarrhea due to decreased risk of any RV infection, not due to decreased severity of infection. A clear pattern of reduced cumulative incidence for any infection was observed in non-secretors compared to secretors throughout the first year of life, suggesting that non-secretors experience fewer primary infections during the most vulnerable risk period of infancy, rather than experiencing similar numbers of primary infections but with reduced severity. The overall pattern also appears to suggest that this decreased risk within the first year may be due to a relative delay in the time to first infection. Similar to RV diarrhea, this appears to be driven primarily in this population by complete protection in non-secretors against infections due to P[4] but not P[8] RVs. This relationship may be population-dependent, as studies in some settings have demonstrated primarily P[8] effects and not P[4], and vice versa [11,22]. Overall, our observations corroborate the P-type distributions that have previously been observed in Bangladesh, where P[8] infections are most common, followed by P[4] and then P[6] [23].

An alternative explanation, suggested by the finding that non-secretors had a significantly higher median Ct value in specimens positive at a very high cut-off of Ct<40, is that non-secretors may experience lower viral burden and clear infections more rapidly compared to secretors. This would mean that we may have missed significantly more asymptomatic infections in non-secretors than in secretors due to a shorter window for detectable stool virus shedding. Previous work within this same cohort has demonstrated that viral burden was significantly reduced in breakthrough diarrhea cases following Rotarix vaccination compared to unvaccinated infants [24]. However, duration of illness was only marginally reduced, and it is unclear if the mechanism for limiting virus shedding due to vaccine-induced immunity is directly translatable to differences observed due to genetically determined factors in unvaccinated infants. 

One other study looked at symptomatic and asymptomatic RV infections at three sites (Bangladesh, Peru, and Tanzania) from the landmark Malnutrition and Enteric Disease Study (MAL-ED) [25]. Of note, the study population from Peru was 100% secretor, and the study population from Bangladesh was recruited from the same urban neighborhood as PROVIDE. Consistent with our findings, secretors were significantly more likely to experience an episode of asymptomatic rotavirus infection through age 2, but curiously, no effect was observed for RV diarrhea, contrary to the preponderance of evidence [25].

Additional clues may be provided by examination of patterns of post-vaccination fecal vaccine shedding [15]. In studies conducted in Brazil, Nicaragua and Malawi, significantly lower frequencies of post-vaccination Rotarix shedding was observed in non-secretors [26,27,28]. Similarly in Malawi, significantly fewer non-secretors shed Rotarix vaccine following the first dose (i.e., primary RV challenge) compared to secretors, but in this study, additional data demonstrate that no differences were observed in peak viral load among vaccine shedders [29]. Interestingly, no difference in vaccine shedding frequency was observed after the second dose. Together, these data support the idea that delay of primary infection may be responsible for a reduction in cumulative incidence of infection during infancy. However, it again remains unclear how comparable these findings, in the context of shedding of a tissue culture-adapted attenuated vaccine strain, are with wild-type asymptomatic infections.

Despite consistent epidemiologic observations, unraveling the molecular basis of the observed P-genotype associations with secretor status remains a work in progress. Unlike human noroviruses, in which infection by certain genogroups is tightly restricted by FUT2 expression [30], FUT2 expression does not appear to be an absolute restriction factor for rotavirus infection, but rather variably limits viral growth [31]. The mechanisms involved remain challenging to elucidate as the pathogenesis of rotavirus infection in traditional in vitro cell culture models appears to be independent of FUT2 [32]. The human intestinal enteroid model provides a useful platform for further evaluation of this phenomenon [33], but presents far more challenges in terms of cost and resource requirements. It is believed that rotavirus attachment to host cells is mediated via binding of the terminal VP8* subunit of the protease-sensitive spike protein VP4 [34]. The VP8* domain from a P[8] virus has been shown to bind to the secretor-dependent H1 antigen at two-fold higher affinity than its non-fucosylated precursor, suggesting a mechanistic basis for increased susceptibility in secretors compared to non-secretors [35]. Interestingly however, studies of human VP8*-binding neutralizing antibodies suggest that the mechanism of neutralization is distinct from the HBGA binding site, obscuring the role of HBGA-virus binding on rotavirus immunity [36,37].

Strengths of this study include the serial longitudinal surveillance specimen collection coupled with extensive community-based surveillance for RV diarrhea incidence, enabling accurate assessments of primary symptomatic vs asymptomatic RV infection. Further, the real-time PCR and sequencing approach provided a sensitive and accurate method for identification of wild-type infections at levels that may have been too low for traditional antigen-based methods. 

There are several limitations to this study. First, while PROVIDE was a comprehensive birth cohort study, this analysis was limited to convenience samples drawn from a subset of participants using a nested case-control selection process primarily determined by RV diarrhea, potentially leading to selection bias. Next, detection of asymptomatic infection relied on a serial cross-sectional design at uneven time intervals, rather than true longitudinal sampling, meaning that it is likely that our detection rate of asymptomatic infections is likely an underestimate. Additional inclusion of serial RV-specific antibody measurements, as has been performed in other RV birth cohort studies [38,39] would have helped mitigate this, but unfortunately serial blood samples were not available for this purpose. We have previously shown that maternal secretor status affects Rotarix seroconversion but not natural infections in unvaccinated infants, likely because maternal effects wane after the early infancy period and may be confounded by associations with maternal antibody levels [40,41]. For these reasons, maternal status was not further evaluated here. Finally, these results may not be generalizable to other settings with different population frequencies of various HBGAs and with different RV epidemiology in terms of P-genotype infection prevalence. For example, it has been well demonstrated that P[6] infections are more frequent in African settings and more highly associated with Lewis, not secretor phenotype [11,22]. 

In conclusion, these data provide additional evidence that non-secretor HBGA phenotype in infants reduces the likelihood of asymptomatic wild-type RV infection, as well as the previously established associations with reduced risk for RV diarrhea. These data further highlight the importance of accounting for host genetic factors in studies of rotavirus disease incidence and vaccine response. Additional research in human populations is needed to understand the mechanisms underlying these findings, which may ultimately help inform the development of next-generation vaccines by illuminating the requirements for RV infection, pathogenesis, and subsequent immune response.

## Figures and Tables

**Table 1:** Selected baseline sociodemographic variables, overall and according to year 1 rotavirus diarrhea status.

		RV Diarrhea in Year 1		
Variable	Total N (%)	Yes N (%)	No N (%)	*p*-Value
Sex at birth				
Female	76 (45%)	38 (50%)	38 (50%)	0.83
Male	93 (55%)	48 (52%)	45 (48%)	
Any household water treatment				
Yes	65 (38%)	38 (59%)	27 (49%)	0.12
No	104 (62%)	48 (46%)	56 (54%)	
Exclusively breastfed at 18 weeks				
Yes	77 (46%)	36 (47%)	41 (53%)	0.34
No	92 (54%)	50 (54%)	42 (46%)	
Stunted at 10 weeks ^1^				
Yes	21 (13%)	10 (48%)	11 (52%)	0.73
No	147 (87%)	76 (52%)	71 (48%)	
Lewis status				
Positive for Lewis a or b	153 (90%)	77 (50%)	76 (50%)	0.65
Negative for Lewis a and b	16 (10%)	9 (56%)	7 (44%)	
Secretor status				
Secretor	114 (67%)	68 (60%)	46 (40%)	0.0001
Non-secretor	55 (33%)	18 (33%)	37 (67%)	

^1^ N = 168 due to missing anthropometric data for one participant.

**Table 2:** Odds of asymptomatic and any rotavirus infection by secretor phenotype.

		Asymptomatic RV Infection				Any RV Infection			
Ct Cut-Off	Phenotype	Yes N (%)	No N (%)	OR ^1^ (95% CI)	*p*-Value	Yes N (%)	No N (%)	OR ^1^ (95% CI)	*p*-Value
<34	Secretor	24 (52%)	22 (48%)			92 (81%)	22 (19%)		
	Non-secretor	4 (11%)	33 (89%)	0.111 (0.034–0.365)	7.4 × 10^−5^	22 (40%)	33 (60%)	0.159 (0.078–0.325)	1.2 × 10^−7^
<40	Secretor	37 (80%)	9 (20%)			105 (92%)	9 (8%)		
	Non-secretor	21 (57%)	16 (43%)	0.319 (0.120–0.848)	0.019	39 (71%)	16 (29%)	0.209 (0.085–0.512)	0.00028

^1^ Odds ratio for infection in non-secretors compared to secretors.

**Table 3:** P-type distribution of rotavirus infections by secretor phenotype.

	Asymptomatic RV Infection			Any RV Infection		
P-Type	Secretor N (%)	Non-Secretor N (%)	*p*-Value	Secretor N (%)	Non-Secretor N (%)	*p*-Value
P[4]	15 (32%)	0 (0%)	0.050	32 (31%)	0 (0%)	9.26 × 10^−5^
P[6]	8 (17%)	1 (9%)	1.00	11 (11%)	3 (8%)	0.76
P[8]	28 (60%)	10 (91%)	0.077	62 (59%)	20 (49%)	0.40

## Data Availability

Data presented in this study are available from the investigators upon request.
